# Protecting children’s health in a calorie-surplus context: Household structure and child growth in the United States

**DOI:** 10.1371/journal.pone.0220802

**Published:** 2019-08-08

**Authors:** Solveig A. Cunningham, Eeshwar K. Chandrasekar, Kate Cartwright, Kathryn M. Yount

**Affiliations:** 1 Hubert Department of Global Health and Department of Sociology, Emory University, Atlanta, Georgia, United States of America; 2 Department of Epidemiology and School of Medicine, Emory University, Atlanta, Georgia, United States of America; 3 School of Public Administration, The University of New Mexico, Albuquerque, New Mexico, United States of America; East Tennessee State University, UNITED STATES

## Abstract

Studies from the social and health sciences have tended to view the household as the locus of access to and distribution of care, resources, monitoring and modeling for children’s wellbeing. Obesity may present a special case for the study of investments in children, being a component of health for which more of certain inputs may not lead to better outcomes. We expanded on common measures of household structure in the child health literature by considering co-residence and relatedness of parents, grandparents, other relatives, and other children. Data were from a longitudinal sample of 6,700 children participating in the Early Childhood Longitudinal Study Kindergarten Class of 1998–99 (ECLS-K), the largest U.S. national dataset with measures of child anthropometrics and household structure at seven time-points over nine years. We used lagged survey-adjusted regressions to estimate associations between household structure and subsequent changes in children’s weight between ages 5 and 14 years in terms of BMI gain and incident obesity. Adjusting for household structure more thoroughly, children living in households with two parents rather than one parent did not experience advantages in terms of less excess weight gain or lower incidence of obesity during elementary and middle school. Children living with a grandmother gained more weight than children not living with a grandmother. Living with siblings and with non-related adults was associated with less weight gain. These findings corroborate a scenario in which, for health problems associated with caloric surplus, classic household factors have more complex associations with child wellbeing.

## Introduction

Obesity tends to develop early in life, with incidence peaking during early childhood.[1,2] The household is the most important context for wellbeing through early and middle childhood; [3] accordingly, expert recommendations identify the household as the most important context in which to prevent or reverse obesity in children.[4–8] Efforts to prevent obesity and to promote healthier weight among children with obesity emphasize family-focused strategies.[4,8–13] However, these efforts generally are agnostic about who in the household matters and how household dynamics can enable or prevent unhealthy growth patterns. Many programs and studies focus on parents, but far fewer consider other co-residing individuals and their roles with respect to children’s risk of obesity. Of these studies, many do not measure the range of household family contexts and their influence on childhood obesity simultaneously.[14–16]

The literature on child health in the social sciences has identified multiple aspects related to household structure, including parents’ marital status, children’s biological relatedness to household decision-makers, and intra-household competition for resources, as critical to children’s wellbeing, because these affect net resources, resource allocation, behavioral modeling, and childcare. Much of this research has been based, theoretically and empirically, in resource-poor contexts, where key relatives can prevent malnutriton by providing much-needed food, or can allow children to attend school by paying school expenses or releasing children from household labor.[17–20]

In this study, we add to the obesity literature theoretically informed, nuanced measures of household structure and apply these to data over nine years from the Early Childhood Longitudinal Study Kindergarten Class of 1998–99 (ECLS-K), the largest nationally representative longitudinal study measuring child wellbeing and household characteristics from Kindergarten through eighth grade. We explore how the conceptulizations of houseold structure and child wellbeing largely developed in resources-poor settings apply to child growth in contexts in which additional calories and less exertion may not be beneficial.

### Household structure and child health

Similarities in bodyweight within families have a genetic component, but behavioral and social factors also are likely to be important, and genetic predispositions work in concert with environmental exposures [3,21–23]. Household structure, typically measured as co-residence with close relatives, relates to several components of child wellbeing, including education, health behaviors, mental and physical health, and growth [3,24–31]. Such relationships likely exist because household structure shapes components of the home environment, including availability and distribution of resources, time allocation, norms and behaviors. Specifically with respect to child growth and the risks of obesity, household structure can affect food availability and eating patterns, levels of activity and inactivity, and rules and supervision, all of which may be linked to children’s risks for obesity [8,32,33].

Contemporary families take many different forms. One aspect important for child wellbeing is the number of co-residing parents: about 65% of American children live with two married biological parents, and this percentage ranges from a low of 32% among African American children to a high of 84% among Asian American children [34]. Over half of children born in the 1990s spent some time in single-parent or step-parent living arrangements [35]. Studies of household structure and children’s wellbeing have focused on the negative consequences of living with one parent, most commonly a single mother [14,30,35,36]. This discussion has centered on the limited economic resources and time to devote to children in these families and the absence or inadequacy of paternal role models and supervision [17,37,38]. In terms of obesity, children living with a single mother more often have been reported to be obese in elementary school than children living with two parents [30,36].

A second less studied component of household structure with respect to child obesity is the parents’ relationship status. Studies from the US and elsewhere indicate that the children of married parents are healthier on a myriad of indicators, beginning with health and survival at birth [39,40]. Also in the US, youths with unmarried parents have had poorer educational outcomes and less health-promoting behaviors than youths living with married parents [35]. Marriage may proxy for the quality and duration of the parents’ relationship, and may commit both partners to invest in children [28,41].

A third aspect of household structure, also understudied with respect to obesity, is the relatedness of co-residing parents to the child. Children living with stepparents are disadvantaged compared with children living with two biological parents [15,41–43]. In the U.S., step-parents have tended to invest less in children than do biological parents: households that include stepchildren spend less on food: spending varies with the relatedness between mother and child, with less spent on adoptive than biological children, less on stepchildren than on adoptive children, and less on foster children than on stepchildren; children living with stepmothers are also less likely to have routine doctor and dentist visits, to have a health care provider, to wear seatbelts, and to live in a non-smoking home [28]. For many components of health, children living with a father and step-mother are no better off than those living only with a father [42,44,45]. Among half-siblings in the same household, those who were step-children to one parent had poorer outcomes in terms of education, health investments and social wellbeing compared to their half-siblings who were biologically related to their parents [15,28,41,44,46,47].

About 15% of American children live in a home with non-parent adult relatives, most often grandparents [48,49]. Children of racial and ethnic minority families are more likely to live with other relatives in addition to or in place of parents: 20%-24% of African Americans, Hispanics, and Asians households include extended family, compared with 9% of non-Hispanic White households [34]. Co-residence with more adults usually has been positively associated with child health because these adults can provide additional supervision and income, though the relatedness of the child to the adult may be relevant [19,27,50–52]. In studies from around the world, children with a surviving nearby or co-resident grandmother are healthier in terms of several indicators, including survival and growth [19,40,50–53]; such relationships were not found for grandfathers [54]. In the US, teenagers living with a single mother and a grandparent had schooling outcomes and health behaviors equal to or better than teenagers living with two parents [35]. The role of non-parent adults is complicated because it often is entangled with parents’ relationship status and living arrangements [55]; still, related adults, especially grandparents, can often mitigate the negative outcomes that children living in non-intact families would otherwise experience [56].

In terms of nutrition and obesity risks, non-parental co-residing adults, especially grandmothers, often assist with childcare, supervision, and the preparation of meals. Literature suggests that children who live with grandparents are more likely to be overweight [57,58]. Grandparents encourage children to eat more, use food as an emotional tool, and favor heavy size in children [16]; mothers have reported pressure from grandmothers, who are concerned about children being too thin, to give children more food, [59]. Children living with grandparents also had poorer weight-related behavior [34,58,60].

Over 75% of American children live with siblings or other children [34]. The relationship between number of children in the household and their health is ambiguous because multiple children entail more competition for resources [44,61,62] but siblings, especially older siblings, also may promote health: in a review, 5 of 6 studies from around the world documented that having older siblings improved survival up to the age of 15 years [54]. In terms of obesity risks, siblings may provide opportunities to be physically active [25]; indeed, children with siblings may be obese less often than single children [36,62,63].

### Implications of household structure in a calorie-surplus context

Previous research has highlighted the ways in which household structure is associated with child wellbeing. Indeed, many of the benefits of household structure for children’s wellbeing have been based in contexts of scarcity–where a single mother may not have the resources to provide sufficient food, clothing, or school fees to promote her child’s health and education; where other adults providing supervision and modeling can facilitate school attendance or vaccination. But what resources are needed to prevent the emerging health problems associated with high energy consumption and inactivity, such as obesity and diabetes? The benefits of household-level investments in more calories and more sedentary time for children’s health in resource-rich settings are less clear than they are in poorer settings: providing maximum nutrition and shielding from physical exertion may actually be detrimental. Observed lower per capita quantities of food in step- and adoptive families may promote healthier weight for children in the U.S. Biological parents and grandparents may be more permissive, buy junk food in response to children’s pleas, not encourage walking commutes, and provide excessive indoor and screen-based games. If so, their children may experience excess weight gain. For obesity prevention and reversal, some family inputs, which in poorer settings would be important investments, for example larger quantities of high-calorie foods or more sedentary time than the local average, could actually lead to *poorer* outcomes. We hypothsize that:

1. Children living in households a) with two parents rather than one and b) with parents who are not biologically related experience lower risks of developing obesity during elementary school;
2. Children living in households with a) additional related or non-related adults or with b) a grandmother have higher risks of developing obesity during elementary school;
3. Children living in households with siblings have lower risks of developing obesity during elementary school than only children.

The relevance of household structure can be assessed in the context of other forces that may be related to child growth. Child characteristics associated with growth patterns include gender, race/ethnicity and age [64–67]: Obesity risks are higher among Hispanic and non-Hispanic black children than among Non-Hispanic white children; these differences emerge early in life [68–70] and continue to grow into adolescence [1]. Low parental education has been correlated with higher rates of child obesity, both because education is associated with employment quality and consequently material (e.g. financial) and immaterial (e.g. time) resources, and because education is associated with greater knowledge about health and nutrition [65,71,72]. These patterns endure across generations, as both parents’ and grandparents’ education is associated with childhood obesity [73]. Compared to households with mothers who do not work, those with working mothers tend to have higher levels of child obesity, as employment can reduce parents’ availability to plan meals and play with children [65,74–76]. Higher rates of obesity have been observed in the U.S. among poorer individuals in recent decades, and poverty may prevent families from providing nutritious food [65,67,71,77]. Children from low-income families are more likely to be obese, even after accounting for parents’ BMI [78]. At age 5 years, children from the poorest quintiles of families had higher obesity prevalence than their peers: 13.8% and 16.5% among the 2 poorest quintiles vs. 7.4% in the wealthiest quintile; obesity prevalence was highest among children in the next-to-poorest quintile, reaching 25.8% by age 14 years [1]. Relevant community characteristics include characteristics of the school attended, urbanicity, and U.S. region of residence. Children who attend public schools have a greater likelihood of obesity than children who attend private schools [79]. There are regional differences in obesity rates in the U.S., with highest prevalence among children in the Southeast and lowest in the Mountain and Western states [80,81]. Childhood obesity rates differ between urban, suburban and rural areas, but these differences tend to be small and vary by age: younger rural children and older urban children are least often obese [81,82].

### Data

The Early Childhood Longitudinal Study, Kindergarten Class of 1998–99 (ECLS-K) is a study of children’s early school experiences, developed by the National Center for Education Statistics and following a large cohort from Kindergarten to 8^th^ grade. Multistage probability sampling was used to select a nationally representative sample of kindergartners [83]. At each wave, measures included direct anthropometric, cognitive and academic assessments and detailed information on the home and school environments. Details on the data have been published elsewhere [84]. Height and weight were measured twice per wave by trained assessors: height in inches to the nearest 0.25 inch using a Shorr Board and weight in pounds using a digital scale [83,85]. This procedure is a major advantage over datasets that collect self-reported or parent-reported anthropometric data, which have been shown to be systematically biased [82,86].

The baseline sample, with 21,260 kindergartners, average age 5.6 years, was collected in the Fall of 1998; subsequent waves were in the Spring of 1999, 2000, 2002, 2004, and 2007 and a 30% sub-sample in the Fall of 1999. Children were retained in the sample if they fell behind or advanced ahead in grades. Most attrition resulted from random selection for non-sampling due to survey costs of children who moved to different schools before fifth grade [83]. With the use of survey weights, the longitudinal sample is representative of individuals who were in Kindergarten in 1998–99 or in 1^st^ grade in 1999–2000.

### Ethics statement

This article is based on the secondary analysis of anonymized, de-identified public-use data files available to researchers via the Inter-University Consortium for Political and Social Research (ICPSR). Human participants were not directly involved in the research reported in this article; therefore, no institutional review board approval was sought.

## Methods

### Variables

The outcome measures used were obesity at each data wave (yes, no), incident obesity among those not obese at the last wave (yes, no) and change in BMI between waves. Obesity in children is more complex to define than in adults because increases in weight and changes in body proportions are part of growth and maturation. Therefore, z-scores or percentiles are typically used for cross-sectional analysis [87]; we constructed BMI z-scores based on weight, height, sex, and age, with reference to the age and sex-specific 2000 CDC Growth Reference with cut-points for obesity at the 95^th^ percentile. For change over time, raw BMI is used because the variability of z-scores over time is lower for the heaviest children [88].

Household structure characteristics were defined at each survey wave in terms of number and relatedness of parents in the household (both biological parents, biological mother only, biological mother and step-father, biological father only, biological father and step-mother, adoptive parents and guardians); parents’ marital status (yes, no); co-resident grandmother (yes, no), other co-residing adult relative (yes, no), co-residing non-relative adult (yes, no); co-residing siblings (none, one or more).

We controlled for child’s sex (male, female), race/ethnicity (non-Hispanic white, black, Hispanic, Asian and Pacific Islander, and Other race including American Indian and multi-racial) and age (years); household socioeconomic status (SES scale divided into quintiles for socio-economic status created by ECLS-K based on parents’ occupational prestige and income [89]); household food-insecurity (yes, no); mother’s employment (full-time, part-time, not employed); type of school currently attended (public, private); U.S. region (Northeast, Midwest, South, West); and urbanicity (urban, large to midsized suburb and large town, small town and rural area).

### Analysis

We used survey adjustments and attrition weights designated by the NCES for all descriptive and analytic methods. We began with descriptive examinations of the data at all waves, with a focus on indicators of body weight and household structure. We then used t-tests to compare characteristics in Kindergarten and eighth grade between children according to their weight status at the end of eighth grade. We used multivariate logistic regressions to identify, at each data wave, which characteristics were associated with obesity.

In longitudinal analyses, we estimated associations between characteristics and circumstances in Kindergarten and subsequent weight status and incident obesity. By focusing on changes over time, we account for the cumulative nature of weight gain over time [86,90]. On the right-hand side are vectors of household structure, child characteristics and household social and economic characteristics, and community characteristics. We used lagged survey-adjusted regressions to estimate the association between household structure and subsequent changes in children’s weight when children were in first, third, fifth, and eighth grade: linear regression with right-hand side variables at time *t* as predictors of weight change between time *t* and *t+1*, and logistic regression with right-hand side variables at time *t* as predictors of incident obesity between time *t* and *t+1*.

## Results

Table 1 shows characteristics of children who attended Kindergarten in the U.S. in 1998 as they grew from Kindergarten through 8th grade. All numbers are rounded to the nearest 10, per NCES requirements. Children’s BMI increased with each wave. The proportion of children who were above the cut-off for obesity was 12% in Kindergarten and increased over time, peaking at 22% in 5^th^ grade.

**Table 1:** Body weight and household characteristics between ages 5 and 14 years among children in the U.S., 1998–2007.

	Kindergarten Fall		1st grade Spring		3rd grade Spring		5th grade Spring		8th grade Spring	
	Mean or percent	SE	Mean or percent	SE	Mean or percent	SE	Mean or percent	SE	Mean or percent	SE
BMI Z-score, M	0.39	(0.03)	0.38	(0.03)	0.54	(0.02)	0.65	(0.02)	0.63	(0.03)
Became obese by next wave (%)	3.59	(0.32)	6.19	(0.52)	5.27	(0.38)	4.03	(0.39)	-	-
Child is obese (%)	11.73	(0.60)	12.66	(0.64)	18.01	(0.72)	21.50	(0.77)	19.27	(0.78)
Number and relatedness of parents in the household (%)										
Both biological parents	68.90	(1.36)	68.33	(1.50)	64.78	(1.36)	62.20	(1.42)	59.12	(1.32)
Biological mother & step-father	6.48	(0.55)	7.61	(0.56)	8.76	(0.64)	9.70	(0.67)	11.69	(0.74)
Biological father & step-mother	1.09	(0.37)	0.77	(0.19)	0.76	(0.16)	1.17	(0.22)	2.08	(0.31)
Biological mother only	17.57	(1.27)	17.97	(1.40)	18.61	(1.18)	20.02	(1.26)	19.16	(1.05)
Biological father only	1.65	(0.35)	1.59	(0.25)	2.25	(0.31)	2.14	(0.30)	2.51	(0.39)
Adoptive parents or guardians	4.30	(0.53)	3.72	(0.37)	4.84	(0.48)	4.75	(0.52)	5.43	(0.52)
Primary caregiver married (%)	71.93	(1.40)	73.06	(1.48)	72.83	(1.33)	71.49	(1.41)	71.89	(1.17)
Co-residing household members										
Grandmother (%)	8.48	(0.62)	10.19	(0.88)	10.97	(0.96)	9.81	(0.90)	8.65	(0.79)
Other adult relative (%)	10.1	(0.01)	7.91	(0.65)	9.21	(0.01)	13.78	(0.89)	14.01	(0.09)
Non-relative adult (%)	4.06	(0.42)	5.88	(0.60)	6.19	(0.53)	5.89	(0.51)	7.29	(0.54)
At least one sibling (%)	82.88	(0.78)	85.99	(0.76)	86.55	(0.72)	87.01	(0.72)	87.87	(0.67)

Data are from the Early Childhood Longitudinal Study Kindergarten Class of 1998–1999. All estimates are survey-adjusted. n = 6,700.

In Kindergarten, 69% of children were living with both biological parents; this percentage decreased over time but remained at above half through 8^th^ grade. The percent of children living with a biological mother (6% in Kindergarten) or biological father (1% in Kindergarten) and step-parent increased over time, as did the percent of children with adoptive or foster parents (4% in Kindergarten). A substantial percentage of children lived with only one biological parent, typically the mother (18% in Kindergarten) and rarely the father (2% in Kindergarten). Over 70% of children had a married mother or primary caregiver at all waves. Across time, about 9% of children lived with a grandmother. One in 10 Kindergartners lived with another adult relative and 4% lived with a non-related adult not designated as a parent; these household types increased substantially by eighth grade (to 14% and 7% respectively). Most children also lived with at least one sibling– 83% in Kindergarten and increasing slightly thereafter.

To understand characteristics that may be associated with excess weight gain during childhood, Table 2 shows the characteristics of Kindergarteners, distinguishing between those who would and would not be obese in 8^th^ grade. Children who were heavier in Kindergarten tended to remain heavy nine years later: among obese children in 8^th^ grade, 67% had been overweight or obese in Kindergarten. Children with obesity in 8^th^ grade more often were boys, children of Hispanic ethnicity, children from lower socioeconomic families, and living in urban areas. Children who were living with their mother and no father in Kindergarten more often were obese in 8^th^ grade, as were children co-residing with their grandmother or another relative; children whose mothers were married in Kindergarten and who had non-relative adults co-residing in Kindergarten were less likely to be obese nine years later.

**Table 2:** Characteristics of children at average age 5 years according to whether they developed obesity by age 14 years.

	No Obesity in 8^th^ grade (n = 5,410)		Obesity in 8^th^ grade (n = 1,290)		p-value
**Characteristics at Kindergarten entry**	**Percent** **/Score**	**Standard Error**	**Percent** **/Score**	**Standard Error**	
**Child characteristics**					
***Sex***					
Male	0.50	(0.01)	0.55	(0.03)	0.08
Female	0.50	(0.01)	0.45	(0.03)	0.08
***Race/ ethnicity***					
White (non-Hispanic)	0.60	(0.02)	0.50	(0.03)	<0.01
Black (non-Hispanic)	0.16	(0.02)	0.19	(0.03)	0.32
Hispanic (black or white)	0.17	(0.02)	0.22	(0.02)	<0.01
Asian	0.03	(0.00)	0.03	(0.00)	0.58
Other race/multiracial	0.04	(0.01)	0.06	(0.02)	0.12
***Type of school attended***					
Public	0.84	(0.01)	0.86	(0.02)	0.35
Private	0.16	(0.01)	0.14	(0.02)	0.35
***Weight status***					
Overweight	0.13	(0.08)	0.23	(0.20)	<0.01
Obese	0.04	(0.04)	0.44	(0.02)	<0.01
Normal weight	0.79	(0.01)	0.32	(0.02)	<0.01
Under weight	0.05	(0.01)	0.01	(0.00)	<0.01
Mean BMI z-score	0.14	(0.03)	1.41	(0.05)	<0.01
**Household characteristics**					
***Socioeconomic status (quintiles)***					
1 (poorest)	0.15	(0.01)	0.23	(0.02)	<0.01
2	0.18	(0.01)	0.22	(0.02)	0.04
3	0.18	(0.01)	0.22	(0.02)	0.07
4	0.23	(0.01)	0.19	(0.02)	0.03
5 (wealthiest)	0.25	(0.01)	0.15	(0.02)	<0.01
***Mother’s Employment***					
No mother in house	0.02	(0.00)	0.02	(0.00)	0.62
Not in labor force	0.27	(0.01)	0.28	(0.02)	0.77
Looking for work	0.03	(0.01)	0.04	(0.01)	0.44
Employed part time	0.23	(0.01)	0.18	(0.02)	0.03
Employed full time	0.45	(0.01)	0.48	(0.03)	0.21
***Food insecurity***^***+***^	0.08	(0.01)	0.10	(0.01)	0.15
***Region of residence***					
Northeast	0.19	(0.01)	0.16	(0.02)	0.06
Midwest	0.25	(0.01)	0.24	(0.03)	0.70
South	0.37	(0.02)	0.41	(0.03)	0.15
West	0.19	(0.01)	0.20	(0.02)	0.72
***Urbanicity***					
Urban	0.35	(0.02)	0.43	(0.03)	<0.01
Midsized town or suburb	0.43	(0.03)	0.36	(0.04)	0.01
Small town or rural	0.21	(0.03)	0.21	(0.03)	0.86
**Household structure**					
***Co-residing parents***					
Both biological parents	0.70	(0.02)	0.67	(0.02)	0.20
Mother and step-father	0.07	(0.01)	0.06	(0.01)	0.53
Father and step-mother	0.01	(0.00)	0.01	(0.01)	0.88
Mother only	0.16	(0.01)	0.21	(0.03)	0.02
Father only	0.02	(0.01)	0.02	(0.01)	0.59
Adoptive parents or guardians	0.04	(0.01)	0.03	(0.01)	0.32
***Primary caregiver is married***	0.74	(0.02)	0.69	(0.03)	0.05
***Grandmother co-resides***	0.08	(0.01)	0.10	(0.01)	<0.01
***Other adult relative co-resides***	0.09	(0.01)	0.11	(0.01)	0.07
***Non-relative co-resides***	0.04	(0.01)	0.02	(0.01)	0.01
***Sibling co-resides***	0.84	(0.01)	0.83	(0.02)	0.61

Data are from the Early Childhood Longitudinal Study Kindergarten Class of 1998–1999

All estimates are survey-adjusted. Some values do not add exactly to 1.00 due to rounding.

P-values calculated by testing linear combinations of parameters (t-tests)

^+^Household food security was assessed by USDA’s 18-item Food Security Module. This variable includes households that reported any food insecurity (with or without hunger) within past 12 months.

Table 3 shows the associations between household structure and children’s obesity through elementary and middle school at each wave, accounting for other characteristics. The patterns indicate that children living with two biological parents do not have lower risks of obesity than children in other living arrangements: after accounting for other characteristics, children living with a biological mother or father and a step-parent had odds of obesity similar to or lower then children living with both biological parents or with just one parent. Children who were adopted had significantly lower odds of obesity in nearly all study waves. Children with a married primary caregiver had equal or lower odds of obesity; however, having accounted for the other household structure and socioeconomic characteristics, there is no additional association with parents’ marital status. Children living with their grandmother had at least equal or higher odds of obesity as children not living with a grandmother. Children living with other relatives had equal or higher odds of obesity, while children living in households with non-relatives tended to have lower odds of obesity, though not significantly so. Children living with at least one sibling had lower odds of obesity than children without siblings.

**Table 3:** Household characteristics and obesity between ages 5 and 14 years: Odds ratios from survey-adjusted logistic regression.

	(1)	(2)	(3)	(4)	(5)
	Obese Fall Kindergarten	Obese Spring 1^st^ grade	Obese Spring 3^rd^ grade	Obese Spring 5^th^ grade	Obese Spring 8^th^ grade
***Co-residing parents*** (Ref = Both biological parents)					
Mother and step-father	0.95	0.81	1.11	0.80	0.85
	(0.19)	(0.18)	(0.24)	(0.20)	(0.18)
Father and step-mother	0.71	0.49	0.54	0.55	0.73
	(0.55)	(0.32)	(0.37)	(0.29)	(0.35)
Mother only	0.63	0.66	0.84	0.86	0.74
	(0.20)	(0.18)	(0.22)	(0.22)	(0.18)
Father only	0.39+	1.24	0.67	0.36*	0.52
	(0.21)	(0.58)	(0.32)	(0.15)	(0.23)
Adoptive parents or guardians	0.43+	0.49*	0.43**	0.57*	0.50**
	(0.15)	(0.17)	(0.13)	(0.15)	(0.12)
***Married***	0.66	0.70	0.96	0.92	0.86
	(0.17)	(0.18)	(0.21)	(0.19)	(0.19)
***Grandmother co-resides***	0.75	1.52*	1.32	1.06	0.98
	(0.18)	(0.25)	(0.24)	(0.21)	(0.22)
***Other adult relative co-resides***	1.41	0.93	1.15	1.26	1.38
	(0.29)	(0.17)	(0.17)	(0.21)	(0.28)
***Non-relative co-resides***	0.62+	0.88	0.73	0.71	0.83
	(0.21)	(0.24)	(0.22)	(0.14)	(0.23)
***At least one sibling co-resides***	0.99	0.87	0.67**	0.85	0.69*
	(0.16)	(0.14)	(0.10)	(0.12)	(0.11)
***Constant***	0.58	0.07+	0.07+	1.37	1.32
	(0.72)	(0.10)	(0.11)	(2.67)	(3.13)

Data are from the Early Childhood Longitudinal Study Kindergarten Class of 1998–1999

All estimates are survey-adjusted. Standard errors are in parentheses.

** p<0.01

* p<0.05

+ p<0.1

Models control for child’s gender, race, age, public or private school attendance, mother’s employment, household’s socioeconomic status quintile, food insecurity, U.S. region, and urbanicity at the indicated survey wave.

In Table 4, we use examine change in BMI over time. The table shows weight change between incremental ages; here, to summarize, we review the long-term patterns of BMI changes between children’s entry into Kindergarten, at average age 5.6 years, and the end of eighth grade, at average age 14.1 years (Model 5). Over this entire period, children living with two biological parents experienced similar BMI increases to children living with a biological parent and a step-parent, with adoptive parents, and with just one parent. Children co-residing with their grandmother gained more body mass than children not co-residing with a grandmother. Children co-residing with other relatives and with non-related adults gained less body mass. Children living with at least one sibling gained significantly less body mass than children without a sibling in the house. Other characteristics associated with high increases in body weight over the nine-year period were being in the lower socioeconomic quintile, living in the South or West U.S., and having a mother who was not working full time.

**Table 4:** Household characteristics and changes in BMI between ages 5 and 14 years.

	(1)	(2)	(3)	(4)	(5)
	Weight change Fall Kindergarten to Spring 1^st^ grade	Weight change Spring 1^st^ to Spring 3^rd^ grade	Weight change Spring 3^rd^ to Spring 5^th^ grade	Weight change Spring 5^th^ to Spring 8^th^ grade	Weight change Fall Kindergarten to Spring 8^th^ grade
***Co-residing parents*** (Ref = Both biological parents)					
Mother and step-father	-0.04	-0.06	0.05	0.09	-0.02
	(0.07)	(0.06)	(0.08)	(0.06)	(0.11)
Father and step-mother	0.49	-0.15	0.07	-0.06	-0.13
	(0.32)	(0.10)	(0.10)	(0.12)	(0.16)
Mother only	0.12*	-0.10*	0.05	0.02	0.04
	(0.06)	(0.07)	(0.05)	(0.07)	(0.08)
Father only	0.34**	-0.11	0.01	-0.17	0.04
	(0.13)	(0.10)	(0.07)	(0.15)	(0.16)
Adoptive parents or guardians	0.02	-0.17**	0.06	0.10	-0.06
	(0.08)	(0.06)	(0.09)	(0.07)	(0.13)
***Married***	0.07	0.05	0.09	-0.01	-0.02
	(0.05)	(0.04)	(0.04)	(0.06)	(0.07)
***Grandmother co-resides***	0.13*	0.08	0.02	-0.06	0.15+
	(0.06)	(0.05)	(0.05)	(0.06)	(0.08)
***Other adult relative co-resides***	-0.05	-0.04	-0.01	-0.02	-0.15*
	(0.05)	(0.06)	(0.04)	(0.05)	(0.07)
***Non-relative co-resides***	-0.01	0.02	0.02	-0.13+	-0.13
	(0.13)	(0.06)	(0.07)	(0.07)	(0.16)
***At least one sibling co-resides***	-0.04	-0.11*	0.00	-0.05	-0.13**
	(0.04)	(0.05)	(0.05)	(0.04)	(0.05)
***Female (ref = male)***	0.02	-0.06*	-0.01	0.11**	0.06+
	(0.03)	(0.02)	(0.02)	(0.02)	(0.04)
***Race (ref = White non-Hispanic)***					
Black (non-Hispanic)	0.00	0.00	-0.01	0.05	0.10
	(0.06)	(0.07)	(0.05)	(0.04)	(0.06)
Hispanic	-0.08+	0.06	-0.04	-0.07	-0.11+
	(0.04)	(0.05)	(0.04)	(0.04)	(0.06)
Asian	-0.02	0.05	-0.09+	0.00	-0.06
	(0.06)	(0.10)	(0.05)	(0.06)	(0.12)
Other Race/Multiracial	-0.13	0.15	0.02	-0.01	0.12
	(0.08)	(0.12)	(0.05)	(0.05)	(0.12)
***Age***	0.06	-0.05	-0.01	0.09**	0.13*
	(0.04)	(0.04)	(0.03)	(0.03)	(0.06)
***Public school***	-0.04	0.04	0.08*	-0.02	0.03
	(0.05)	(0.03)	(0.03)	(0.03)	(0.06)
***Mother working full-time***	-0.08*	0.05+	-0.06**	0.00	-0.10*
	(0.03)	(0.03)	(0.02)	(0.03)	(0.04)
***SES Quintile (ref = poorest)***					
Second Quintile	0.02	-0.02	0.07	0.01	-0.13+
	(0.05)	(0.06)	(0.07)	(0.04)	(0.07)
Third Quintile	-0.06	0.01	0.04	-0.06	-0.17*
	(0.05)	(0.06)	(0.05)	(0.06)	(0.07)
Fourth Quintile	-0.08	-0.02	0.01	-0.07	-0.21**
	(0.06)	(0.06)	(0.07)	(0.05)	(0.07)
Fifth Quintile	-0.06	-0.06	-0.03	-0.06	-0.28**
	(0.06)	(0.06)	(0.07)	(0.05)	(0.07)
***Food insecurity***	0.05	0.03	0.07+	0.02	0.03
	(0.06)	(0.06)	(0.04)	(0.04)	(0.07)
***Region (ref = Northeast)***					
Midwest	-0.05	0.04	0.01	0.05+	0.06
	(0.05)	(0.05)	(0.03)	(0.03)	(0.06)
South	0.03	0.03	0.06	0.01	0.12*
	(0.04)	(0.05)	(0.04)	(0.04)	(0.05)
West	0.03	0.05	-0.01	0.02	0.15*
	(0.05)	(0.05)	(0.04)	(0.05)	(0.07)
***Urbanicity (ref = Central City)***					
Midsized or large suburb	0.00	-0.02	-0.02	-0.03	-0.04
	(0.04)	(0.04)	(0.03)	(0.03)	(0.05)
Small town or rural	-0.03	0.03	-0.05	-0.05+	-0.05
	(0.05)	(0.05)	(0.03)	(0.03)	(0.07)
***Constant***	-0.28	0.50*	0.13	-0.87*	-0.28
	(0.26)	(0.25)	(0.29)	(0.35)	(0.34)

Data are from the Early Childhood Longitudinal Study Kindergarten Class of 1998–1999

All estimates are survey-adjusted. Standard errors are in parentheses

** p<0.01

* p<0.05

+ p<0.1

Table 5 shows the risks of incident obesity among children not already obese at each data wave and over the entire period between Kindergarten entry and end of eighth grade. The patterns are consistent with those observed in BMI change, but notably there are no significant differences in long-term odds of incident obesity, that is, crossing over the 95^th^ percentile, associated with household structure.

**Table 5:** Household structure and odds of incident obesity between ages 5 and 14 years.

	(1)	(2)	(3)	(4)	(5)
	Incident obesity Fall Kindergarten to Spring 1^st^ grade	Incident obesity Spring 1^st^ to Spring 3^rd^ grade	Incident obesity Spring 3^rd^ to Spring 5^th^ grade	Incident obesity Spring 5^th^ to Spring 8^rd^ grade	Incident obesity Fall Kindergarten to Spring 8^th^ grade
***Co-residing parents*** (Ref = Both biological parents)					
Mother and step-father	1.03	1.86+	0.99	0.86	0.93
	(0.41)	(0.62)	(0.42)	(0.47)	(0.27)
Father and step-mother	1.59	1.31	1.42	1.13	-
	(1.60)	(1.37)	(1.50)	(1.31)	
Mother only	1.11	1.53	1.00	0.61	1.26
	(0.31)	(0.57)	(0.42)	(0.38)	(0.42)
Father only	1.54	1.61	0.52	0.34	0.61
	(1.08)	(1.21)	(0.30)	(0.30)	(0.33)
Adoptive parents or guardians	0.82	0.76	0.90	0.43	0.74
	(0.40)	(0.36)	(0.47)	(0.39)	(0.37)
***Married***	1.02	2.07	0.89	0.64	1.13
	(0.22)	(0.67)	(0.31)	(0.34)	(0.31)
***Grandmother co-resides***	2.71**	1.22	0.93	1.61	1.60
	(0.83)	(0.35)	(0.29)	(0.71)	(0.48)
***Other adult relative co-resides***	0.47*	1.09	0.87	0.67	0.76
	(0.16)	(0.28)	(0.23)	(0.24)	(0.18)
***Nonrelative co-resides***	0.38*	1.04	0.45	0.07**	0.66
	(0.19)	(0.50)	(0.22)	(0.06)	(0.30)
***At least one sibling co-resides***	0.74	0.54**	1.28	1.69+	1.02
	(0.19)	(0.11)	(0.38)	(0.49)	(0.18)
***Constant***	0.00**	0.02*	5.63	0.04	0.19
	(0.00)	(0.04)	(16.78)	(0.15)	(0.24)

Data are from the Early Childhood Longitudinal Study Kindergarten Class of 1998–1999

All estimates are survey-adjusted. Standard errors are in parentheses

** p<0.01

* p<0.05

+ p<0.1

Models control for child’s gender, race, age, public or private school attendance, mother’s employment, household’s socioeconomic status quintile, food insecurity, U.S. region, and urbanicity for years 1998–2007.

## Discussion

Previous studies have shown that household structure is associated with children’s wellbeing, including health at birth, survival, growth, and education. Specifically, around the world, children whose parents are in married or long-term relationships, who are more closely related to co-residing adults, and who live with adults in addition to parents, tend to have better outcomes, primarily because of greater investments in resources such as food, vaccination, and school expenses [8,40,54]. The relationships between household structure and children’s excess weight gain and risk of obesity requires a clearer understanding to better guide recommendations [8,63,91]. This relationship has grown in importance as obesity becomes increasingly prevalent worldwide and is especially relevant today in settings like the U.S., where absolute caloric scarcity is not a widespread challenge.

Our findings indicate that one of the major health concerns for children today, obesity, may not be associated in the same way with household structure as are other aspects of child wellbeing. Adjusting for household structure more thoroughly than studies typically have, children living in households with two parents rather than one parent did not experience advantages in terms of lower weight gain or lower obesity risks during elementary and middle school. A prior study using the same dataset in fifth grade reported that children living with single mothers were more likely to be obese than children living with two parents [36,63]. We replicated these findings, but this relationship may be driven by differences between children living with two biological parents and those living with parents who were not their biological parents. Thus, we found no significant evidence of different growth patterns and obesity risks between children living with two parents or one.

Adjusting for other characteristics of households, relatedness of the parents to the children was not associated with children’s growth. Children living with a parent and a step-parent experienced similar or lower BMI gain and incident obesity to those living with a single parent and those living with adoptive parents. There is some indication that children living without their biological mother, including those living with adoptive parents or only with their biological father had lower risks of developing obesity. Previous studies have found that the mother is the most important relative promoting child health and that children living without their mother were at significantly higher risk of mortality and other negative outcomes, at least in part because they receive fewer resources and less care [42,44]. Studies have shown that step-mothers do not substitute for mothers [15,43], and our findings corroborate these reports, but paradoxically indicate that living with a step-parent is not associated with worse outcomes in the case of obesity. One concern would be whether the absence of obesity is actually a negative outcome, in that it could indicate underweight, but we did not find this to be the case, as children living in step-families were not more often underweight than children living with both biological parents.

Previous studies have highlighted the protective role of grandmothers in child wellbeing, but also have indicated that they may promote obesity. In this study, children living with a grandmother had more increases in BMI z-scores and were more likely to develop obesity than children not living with a grandmother.

Living with siblings was associated with lower obesity risks and lower weight increases. Thus, sibling competition for resources does not lead to poorer outcomes in terms of obesity. Perhaps parents with multiple children provide more structured lifestyles for financial or organizational reasons [61], and these lifestyle differences are protective against excessive weight gain [36,92]. It may also be that children living together with siblings or other youths have more opportunities for active play [25,26]. Also, single children may have more influence over their parents in choosing their own activities and health-relevant behaviors [62].

Household structure, socioeconomic status, and investments are intertwined attributes of a child’s environment. In the U.S. and other Western countries, children from poorer families are often are more likely to develop obesity [93–96]. In this cohort, at age 5, the prevalence of obesity was highest among children from the poorest 40% of families; across elementary school, it was highest among children in the next-to-poorest quintile, reaching 25.8% by age 14 years [1]. At the same time, having accounted for economic resources and multiple other characteristics, it may be that household structure arrangements that facilitate even more resources do not have healthier child weight in an environment where the most common nutrition-related health problem is not underweight but obesity. Children living with step-parents have been shown in other studies, including from the U.S., to have lower access to resources, including resources pertinent to health [28,42,44,45,47]. In this study, we found that they do not gain more weight during elementary school or have higher risks of incident obesity in childhood. Grandmothers, who often provide either additional economic resources or care and supervision for children, do not improve outcomes in the domain of body weight. Similarly, having more children in the home, which may decrease per capita resources, nonetheless was associated with less weight gain and obesity.

A limitation of this study is that we could not take into account the quality of interactions among household members or the duration of parents’ relationships, which are likely to be pertinent to child wellbeing. Some children are in dual custody arrangements, spending some of their time in multiple households, and we have only accounted for the structure of the household where they spend the most time. We did not distinguish children living with same-sex parents. We also did not identify which household interactions and activities are associated with weight trajectories. A recent study using the same longitudinal cohort showed that, after accounting for weight at younger ages, standard behavioral factors -intake of fruit, vegetables, fast food and soda, television viewing, and physical activity–individually had little or no effect on subsequent weight [97]. This pattern indicates that future studies should take a comprehensive approach to measuring the behavioral links between household structure, home environment and children’s weight.

The practical implications of these findings raise questions about household investment strategies in a world of caloric abundance. Historically, across cultures, a crux of households investments to promote child wellbeing have been to provide abundant, tasty food and to protect children from caloric depletion and exhaustion by reducing the amount of heavy work or exhausting travel. It may be that these strategies perpetuated in the contemporary world entail the provision of excessive caloric intake through large portions of palatable food and insufficient caloric expenditure through safe, yet sedentary leisure and learning, often in front of a screen. These strategies may continue to signify care but may not be protective against excess weight gain in childhood and obesity.
